# Purification and preliminary crystallization of alanine racemase from *Streptococcus pneumoniae*

**DOI:** 10.1186/1471-2180-7-40

**Published:** 2007-05-17

**Authors:** Ulrich Strych, Milya Davlieva, Joseph P Longtin, Eileen L Murphy, Hookang Im, Michael J Benedik, Kurt L Krause

**Affiliations:** 1 University of Houston, Department of Biology and Biochemistry, 4800 Calhoun Rd, Houston, TX 77204-5001, USA; 2 Department of Biology, Texas A&M University, College Station, TX 77843-3258, USA; 3 Department of Biochemistry, University of Otago, P.O. Box 56, Dunedin, New Zealand

## Abstract

**Background:**

Over the past fifteen years, antibiotic resistance in the Gram-positive opportunistic human pathogen *Streptococcus pneumoniae *has significantly increased. Clinical isolates from patients with community-acquired pneumonia or otitis media often display resistance to two or more antibiotics. Given the need for new therapeutics, we intend to investigate enzymes of cell wall biosynthesis as novel drug targets. Alanine racemase, a ubiquitous enzyme among bacteria and absent in humans, provides the essential cell wall precursor, D-alanine, which forms part of the tetrapeptide crosslinking the peptidoglycan layer.

**Results:**

The alanine racemases gene from *S. pneumoniae *(*alr*_*SP*_) was amplified by PCR and cloned and expressed in *Escherichia coli*. The 367 amino acid, 39854 Da dimeric enzyme was purified to electrophoretic homogeneity and preliminary crystals were obtained. Racemic activity was demonstrated through complementation of an *alr *auxotroph of *E. coli *growing on L-alanine. In an alanine racemases photometric assay, specific activities of 87.0 and 84.8 U mg^-1 ^were determined for the conversion of D- to L-alanine and L- to D-alanine, respectively.

**Conclusion:**

We have isolated and characterized the alanine racemase gene from the opportunistic human pathogen *S. pneumoniae*. The enzyme shows sufficient homology with other alanine racemases to allow its integration into our ongoing structure-based drug design project.

## Background

*Streptococcus pneumoniae *is a Gram positive diplococcus that colonizes the upper respiratory tract in about 20% of healthy humans, but is a leading cause of diseases such as otitis media, bacteremia, lobar pneumonia, and meningitis [1], particularly when enclosed in type-specific polysaccharide capsules. Among the elderly, *S. pneumoniae *is the most common cause of fatal community-acquired pneumonia [2], and in children the most common cause of community-acquired pneumonia, middle ear infections and meningitis [3]. From 1970 to 1990, resistance to *S. pneumoniae *has increased significantly, presumably due to the increased use of antibiotics. The first reports of penicillin-resistant *S. pneumoniae *strains appeared in the US in the early-1980s [4], then beginning in the 1990s their share increased from less than 5% of all isolates to approximately 35% in 2002 [5]. Similarly, resistance to macrolides is increasing, with 24% of all isolates being resistant in 2000 [6]. To further complicate the issue, multidrug resistance is increasingly present among *S. pneumoniae *isolates, documented by a recent study showing 22 % of all isolates to be resistant to at least three antibiotics [7].

In light of this growing resistance problem, the identification and characterization of more potential targets for antibiotic therapy is of paramount importance. The inhibition of bacterial growth through antibiotics targeting cell wall biosynthesis has been a proven mode of action since the beginning of the antibiotic era [8]. While penicillin targets the crosslinking reaction in peptidoglycan biosynthesis, there are also choices when it comes to inhibiting the formation of essential precursors for the peptidoglycan layer. One of those precursors, the amino acid D-alanine, is an essential component of the tetrapeptide crosslinking the glycan strands. It is provided through the racemization of the naturally occurring L-alanine, catalyzed by the cytoplasmic enzyme alanine racemase (Alr, EC 5.1.1.1.).

Alr is a pyridoxal 5'-phosphate containing enzyme, ubiquitous among most bacteria, and absent in humans. A lysine residue connected to the PLP cofactor by an internal aldimine bond acts as a base for the conversion of D-to L-alanine while a nearby tyrosine from the second monomer acts as a base for the abstraction of a hydrogen from L-alanine [9]. We have determined crystal structures of alanine racemases from other human pathogens in the past. Notably, the enzymes from *Mycobacterium tuberculosis *and *Pseudomonas aeruginosa *have been resolved at high resolution [10,11]. Both enzymes are dimers with a similar domain makeup and include both an α/β-barrel at the N-terminus and a C-terminus primarily made of β-strands. Because the active site of Alr is small we are interested in identifying conserved residues that regulate the entrance of the substrate into the substrate binding pocket [11] with the goal of developing compounds to control access of substrate to the active site.

This paper describes the molecular cloning, expression, purification, and elucidation of the biochemical properties of the alanine racemase from *S. pneumoniae*. We show evidence that the *alr*_*SP *_gene encodes a functional alanine racemase through complementation of an *E. coli *D-alanine auxotroph, and through a specific spectrophotometric assay. We have obtained preliminary crystals of *S. pneumoniae *Alr, and intend to incorporate the enzyme into our ongoing structure-based drug design program [12,13]. Determining the structure of Alr_SP _is an essential prerequisite for the development of an accurate pharmacophore model of the enzyme. Using our structure will allow us to conduct molecular dynamics simulations to obtain information about the different conformations of the enzyme (and its substrate), eventually yielding a dynamic pharmacophore model. Using *in silico *methods, compounds from the Available Chemicals Directory [14] that fit the pharmacophore model will be identified and submitted to experimental testing, first *in vitro *and subsequently *in vivo*. Proof of principle has been provided by our collaborators at the University of Houston for the alanine racemase from *G. stearothermophilus *[15].

## Results and discussion

### Amplification and cloning of the *S. pneumoniae *alr gene

The putative open reading frame for the alanine racemase from *S. pneumoniae *was identified through sequence comparison between known alanine racemase sequences and the *S. pneumoniae *sequences deposited in GenBank. There was only one unambiguous hit in the database (data not shown), suggesting that *S. pneumoniae*, unlike *E. coli *or *P. aeruginosa*, contains only one alanine racemase. The second alanine racemase (DadX) when present is commonly part of an operon. The *S. pneumoniae *genome carries only this single gene, not apparently in an operon, and thus is lacking the L-alanine inducible catabolic DadX alanine racemase. Using primers homologous to the 5' and 3' end of the putative *alr*_*SP *_an 1103 bp fragment was amplified and subsequently sequenced. The *orf *encodes a polypeptide of 367 amino acids with a calculated molecular weight of 39854 Daltons. Using the restriction sites incorporated into the PCR primers the gene was ligated into the expression vector gene pET17 and cloned in *E. coli *MB1547.

### Sequence analysis of Alr

**Figure 1:**
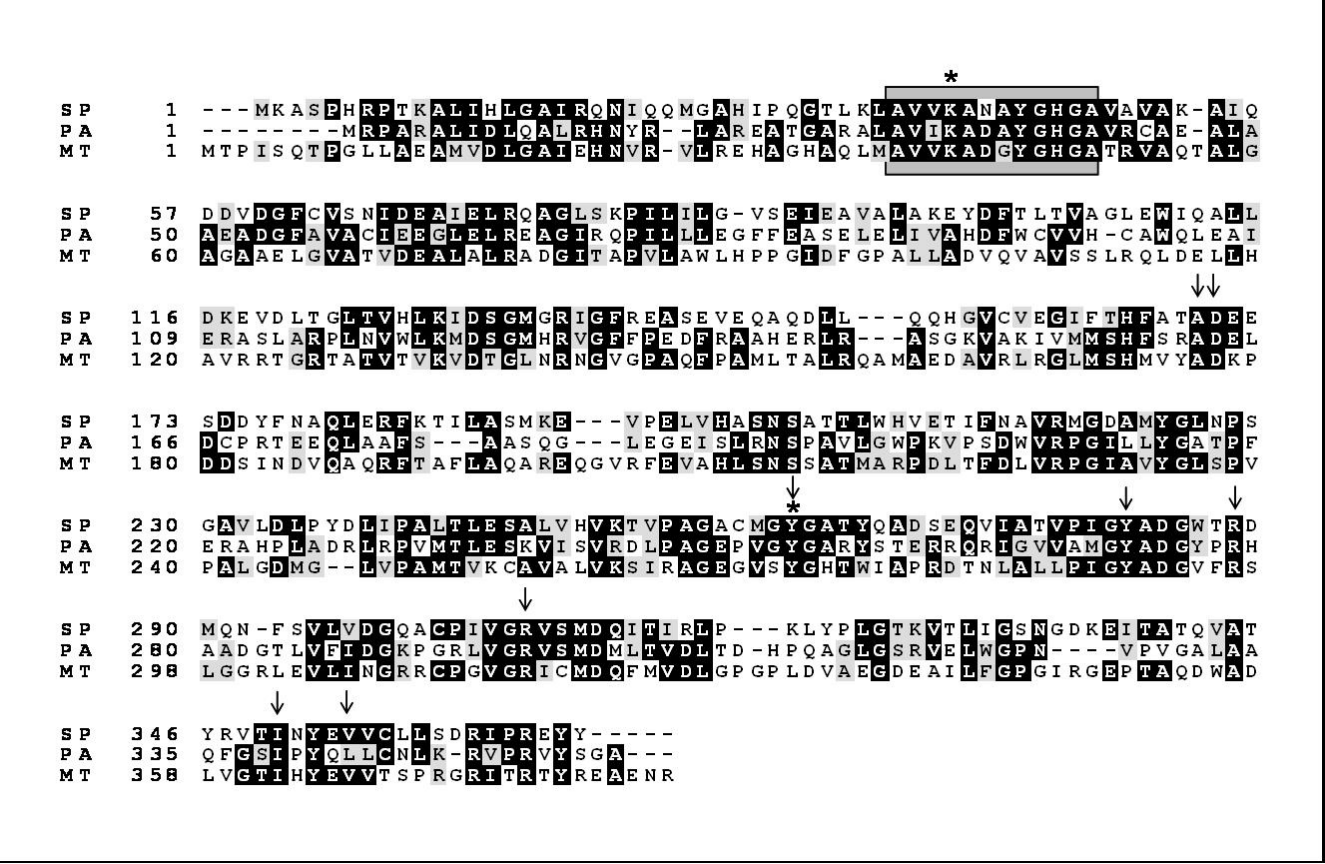
ClustalW multiple sequence alignment of alanine racemases from *S. pneumoniae *(SP), *P. aeruginosa *DadX (PA) and *M. tuberculosis *(MT). The conserved pyridoxal 5'-phosphate binding site is boxed. An asterisk (*) is used to indicate the two catalytic residues, K40 and Y263. Arrows indicate eight conserved amino acid residues constituting the entryway to the active site [11].

**Figure 2:**
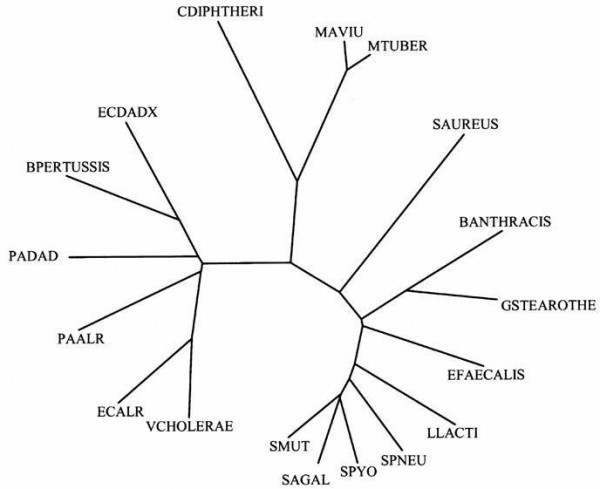
Phylogenetic tree of ALR based on amino acid sequences. The tree was constructed using the ClustalW and Phylip programs as described in Section 2.x.: VCHOLERAE (*Vibrio cholerae*, NP230026), ECALR (*E. coli*, NP418477), PAALR (*Pseudomonas aeruginosa*, AAD47082), PADADX (*P. aeruginosa*, AAD47081), ECDADX (*E. coli*, NP415708), BPERTUSSIS (*Bordetella pertussis*, NP879994), CDIPHTHERI (*Corynebacterium diphtheriae*, NP938944), MAVIUM (*M. avium*, AAF25943), MTUBER (*M. tuberculosis*, AAD51033), BANTHRACIS (*Bacillus anthracis*, NP842805), GSTEAROTHE (*Geobacillus stearothermophilus*, P10724), EFAECALIS (*Enterococcus faecalis*, NP814591), LLACTIS (*Lactobacillus lactis*, NP_267000), SPNEU (*S. pneumoniae*, AAD51027), SPYO (*S. pyogenes*, YP603085), SAGAL (*S. agalactiae*, NP688675), SMUT (*S. mutans*, NP722151).

Comparison and analysis of the protein sequence encoded by *alr*_*sp *_from strain R800 revealed full identity with the protein sequences from strains R6 (GenBank accession number: P0A2W9) and TIGR4 (GenBank accession number: AAK75776). Moreover, it displayed a high level of similarity with other alanine racemases (Fig. 1), and carried the expected motifs such as the characteristic pyridoxal phosphate binding site (VVKANAYGHG) near the N-terminus, and the two catalytic amino acid residues (K40, Y263) of the active center [11]. Phylogenetic analysis (Fig. 2) revealed no surprises; the enzyme is clustered with other Streptococci species, such as *S. mutans *or *S. pyogenes*. These sequences, together with other alanine racemases from Gram-positive bacteria appear to occupy one half of the phylogenetic tree, while the Gram-negative bacteria occupy the other half.

Since both alanine racemase and certain human enzymes, such as serine racemase [16] depend on pyridoxal-5'phosphate (PLP) as their co-factor, it is important that antibiotic design efforts aim to develop inhibitors that are specific for the bacterial enzyme. However, it is worth noting that, in the case of serine racemase and Alr_SP _both enzymes are indeed significantly distinct. The two racemases are only 13% identical on the amino acid level, so while they both *may *still be inhibited by the same small molecule, there is significant diversity between the enzymes, making it more likely that a potential inhibitor will actually be alanine racemase specific. In addition, our drug design efforts extend well beyond the PLP binding site of the enzyme. As shown in our previous publication [11], alanine racemases are characterized by a geometrically distinct entryway to the active site that is made up of eight highly conserved amino acid residues. This entryway is fully conserved in the Alr_SP _sequence; residues Y253, Y352, Y282 and A169 form the inner layer, while residues R307, I350, R288 and A170 constitute the middle layer. Sequence analysis of the human serine racemase fails to identify those conserved residues. We thus hypothesize that an inhibitor designed to block the entryway would specifically inhibit the bacterial target, alanine racemase, and be much less likely to cross-react with eukaryotic PLP-containing enzymes.

### Complementation analysis

*In vivo *demonstration that the gene product expressed alanine racemase activity was shown by complementation. The D-alanine auxotrophic *E. coli *strain MB2795 was transformed with pET17-*alr*_*SP*_. A plasmid encoding the cloned *P. aeruginosa *DadX alanine racemase, pMB1921 was used as a positive control. pET17 without insert served as a negative control. Cells were plated on LB medium with and without D-alanine supplementation, and scored for colony growth after 16 h at 37°C. The alanine racemase gene from *S. pneumoniae *fully restored the wild-type phenotype, as did the *P. aeruginosa dadX *plasmid. Cells transformed with pET17 failed to grow. Thus the cloned *S. pneumoniae *gene encodes a functional alanine racemase.

### Overexpression, purification and biochemical characterization

**Figure 3:**
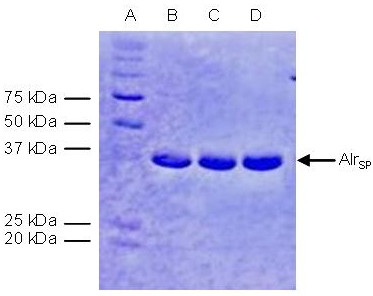
SDS-polyacrylamide gel electrophoresis of alanine racemase from *S. pneumoniae *(Lanes B-D) and protein molecular weight markers (Lane A, BioRad Dual Color Marker) stained with Coomassie blue. Lanes B-D represent the three peak fractions from the final gel filtration step.

The recombinant pET17-*alr*_*SP *_plasmid was transformed into *E. coli *BL21(DE3), pLysS, and expressed upon induction of T7 polymerase. The enzyme was purified to electrophoretic homogeneity as summarized in Table 1. Purified enzyme was obtained in an overall yield of 28.7% and exhibited a 12.9 fold increase in specific activity over the crude lysate. The molecular mass of the purified protein, estimated by SDS-PAGE analysis, was approximately 39 kDa, in line with the calculated value for the *alr*_*SP *_open reading frame (Fig. 3). The kinetic properties of the purified *S. pneumoniae *enzyme are similar to other alanine racemases, it has a *K*_*m *_for D-alanine at 23°C of 2.10 mM and for L-alanine of 1.92 mM. The *V*_*max *_for the racemization (D- to L-alanine and L- to D-alanine) is 87.0 and 84.8 U mg^-1^, respectively, where one unit was defined as the amount of enzyme that catalyzed racemization of 1 μmol of substrate per minute. These values were used to calculate a *K*_*eq *_of 1.07 for this reaction, thus fulfilling the criterion (*K*_*eq*_~1.0) for a chemically symmetric reaction [17].

Some recent data suggested that alanine racemases are either monomeric or dimeric [18]. Initially, molecular sieve chromatography was used to answer this question for Alr_SP_. The molecular weight of five protein standards was plotted versus the ratio of their elution volume to the void volume of the column to yield a linear calibration curve. This curve was then used to determine an apparent molecular weight of 80000 Daltons for Alr_SP_, a value suggesting a dimeric state for the enzyme. In order to further classify Alr_SP _we additionally performed dynamic light scattering using the Superdex purified enzyme. When tested at 1.8 mg/ml we observed a single peak with a hydrodynamic radius of 3.7 nm and a monodisperse profile. This radius corresponds to a molecular weight of 71000 Daltons, clearly suggesting that in solution the dominant form of Alr_SP _is the dimer. Even at lower concentrations of protein, we were not able to see any evidence for Alr_SP _monomers, nor did we observe any mixed populations of monomers and dimers in the same preparation. As a result we conclude that this enzyme belongs to the majority of alanine racemases that form dimers, as do the enzymes from *M. tuberculosis *[11] and *P. aeruginosa *[19].

### Preliminary crystallization

**Figure 4:**
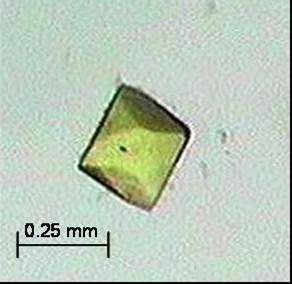
Alr_SP _crystal (0.25 × 0.25 × 0.1 mm), obtained in a solution of 1.2 M NaCitrate, 0.1 M MES, pH7.2, 10% glycerol, by using the sitting vapor diffusion method.

Preliminary crystals for Alr_SP _were obtained using a sparse matrix crystallization approach [20]. Initial crystals were obtained in 1.4 M NaCitrate, 0.1 M Hepes, pH7.5. This condition was successively modified and better crystals of 0.25 × 0.25 × 0.1 mm were eventually obtained in 1.2 M NaCitrate, 0.1 M MES, pH7.2, 10% glycerol (Fig. 4). Data analysis using X-ray crystallography is underway.

## Conclusion

We have isolated the gene encoding alanine racemase from *S. pneumoniae *and obtained high level heterologous expression in *E. coli *as a dimer of 80 kda. Sequence homology, complementation of a D-alanine auxotroph, and a specific spectrophotometric enzyme assay confirm the identity of the cloned protein. With the recent acquisition of protein crystals described here, and the homology with other known alanine racemases whose structures are available, we anticipate the structure of this enzyme should soon be available, allowing it to be integrated into our ongoing structure-based drug design project.

## Methods

### Bacterial strains, plasmids and culture conditions

*E. coli *MB1547 (*supE thi hsdΔ5 Δ(lac-proAB) endA *F'[*traD36 proAB lacI*^*q*^*lacZΔM15*]) was used for routine cloning and molecular biology. *E. coli *MB2795 is an alanine racemase deficient double-mutant (*alr::frt dadX::frt*) of *E. coli *MG1655 that we routinely use to assess alanine racemase activity of cloned fragments from bacteria [21]. Overexpression of alanine racemase from *S. pneumoniae *was performed in *E. coli *BL21 (DE3), pLysS (Novagen). pET17 (Novagen) was the vector used for expression of the *alr *gene from *S. pneumoniae*. pMB1921, expressing the alanine racemase (DadX) from *Pseudomonas aeruginosa *[22], was used as a positive control in the complementation experiments. All cells were routinely grown in LB medium with 100 μg/ml ampicillin. *E. coli *BL21 (DE3), pLysS cultures also contained 30 μg/ml chloramphenicol.

### Gene cloning

Plasmid pR283 [23] derived from *S. pneumoniae *R800 was used as template for PCR amplification. Two primers (5' CCCATATGAAAGCTAGTCCA CATAGACCAACC 3', 5' GGGGATCCTTTCTTTTCTAATAATATTCTCTCGG 3', underlined sequences represent *Nde*I and *Bam*HI restriction sites, respectively) were designed based on sequence comparisons between known alanine racemase genes and the *S. pneumoniae *R6 sequence in GenBank [24]. Gene amplification was performed as described [22]. The PCR product was digested with *Nde*I and *Bam*HI and ligated into an appropriately cut pET17 vector, yielding pET17-*alr*_*SP*_.

### Nucleotide sequencing

DNA sequencing was performed on an ABI Prism 373 sequencer using the dye terminator cycle sequencing kit (Perkin-Elmer). Primers complementary to the T7 promoter and terminator regions were used to determine the sequence of the cloned fragment.

### Sequence analysis

Nucleotide sequence comparisons were conducted at the NCBI website [25] using the Blast algorithm [26]. Multiple sequence alignments were performed using ClustalW [27] at the European Bioinformatics Institute [28] website. Phylogenetic relationships were investigated using the Phylip software package [29] at the Pasteur Institute [30].

### Protein expression and purification

*E. coli *BL21(DE3), harboring pET17-*alr*_*SP *_pLysS was grown, induced and harvested as described [22]. The cell pellet from 6 l of culture was initially suspended in 90 ml 20 mM Tris-HCl, pH8, 0.5 mM pyridoxal phosphate. After sonication (6 × 50 W for 20 sec) and centrifugation (JA20, 27000 × g, 25 min), ammonium sulfate ((NH_4_)_2_SO_4_)) was added to the supernatant to a final concentration of 20% saturation. The precipitate was removed by centrifugation (as above), and (NH_4_)_2_SO_4 _was added to the supernatant to a final concentration of 60% saturation. After centrifugation (as above) the pellet was suspended in 35 ml 20 mM Tris-HCl, pH8, dialyzed against 20 mM Tris-HCl, pH8, and purified over a Q-Sepharose High Performance column (GE Amersham) using a 0–1 M NaCl gradient in 20 mM Tris-HCl, pH8. The peak fractions were pooled, (NH_4_)_2_SO_4 _was added to 1 M, and the sample was further purified through HiPrep Phenyl (low sub) hydrophobic interaction chromatography (GE Amersham) using a 1-0 M (NH_4_)_2_SO_4 _gradient. The peak fractions were pooled, dialyzed against 20 mM Tris-HCl, pH8, concentrated and loaded onto a Superdex 200 Prep Grade column (GE Amersham) for the final purification step and peak fractions were pooled and retained.

### Enzyme assay, characterization, and protein determination

Throughout the purification quantity and quality of the preparation were monitored by assaying protein concentration [31] and enzyme activity [22]. The purification was further monitored using SDS-PAGE on a GE-Amersham PHAST gel system. The molecular weight of the native enzyme was estimated by comparing its migration on the Superdex 200 column with five protein standards (12–200 kDa) from the Sigma MWGF200 standards kit. Dynamic light scattering of a 1 mg/ml sample of Alr_SP _was done at 20°C using the DynaPro Titan system according to the manufacturer's instructions (Wyatt Technology). Prior to the measurements, all samples were spun in an Eppendorf 5415 tabletop centrifuge and then filtered through a 0.02 micron Whatman *Anotop *filter in order to remove aggregates prior to measurement of light scattering data.

### Crystallization

Alr_SP _was crystallized at a concentration of 21 mg/ml using the sitting drop vapor diffusion method. 1 μl of protein and 1 μl of mother liquor were automatically mixed in a 96 well plate using the Honeybee 961 crystallization robot (Genomic Solutions). Each plate was set up at 4°C with the 96 conditions of the Crystal Screen HR2-130 kit (Hampton Research). Image analysis was done by visual inspection.

### Sequence Accession Numbers

The nucleotide and amino acid sequences of ALR_SP _have been submitted to GenBank under Accession Nos. AF171873 and AAD51027, respectively.

## Authors' contributions

US was responsible for cloning and, in part, expression and biochemical characterization of the alanine racemase, and prepared the manuscript. MD and JLP performed the expression and purification of ALR, as well as the light-scattering experiments. EM and HI performed crystallization experiments. MJB and KLK supervised the work, organized financial support, and critically appraised the manuscript. All authors read and approved the final manuscript.

## Figures and Tables

**Table 1:** Purification of alanine racemase from *Streptococcus pneumoniae*

	Volume [ml]	Protein Conc [mg/ml]	Total Protein [mg]	Specific Activity [u/mg]	Total Activity [u]
Crude Extract	90.00	12.50	1125.00	5.10	5736.54
Supernatant after 20 % AmSO_4 _precipitation	95.00	8.90	845.50	6.33	5358.91
Resuspended pellet after 60 % AmSO_4 _precipitation	35.00	22.50	787.50	6.74	5311.40
Before Q-Sepharose	45.00	13.00	585.00	8.16	5143.09
After Q-Sepharose	24.00	3.80	91.20	45.87	4174.49
After HIC	10.00	7.00	70.00	49.91	3556.27
Before Superdex	8.00	3.30	26.40	64.09	1692.09
After Superdex	0.50	50.00	25.00	65.81	1645.23
